# Bilateral Vocal Cord Palsy Secondary to Rheumatoid Arthritis

**DOI:** 10.7759/cureus.51958

**Published:** 2024-01-09

**Authors:** Liam D Hyland, Rachel Saville, Nada Dwiddar, Milind Sovani

**Affiliations:** 1 Otolaryngology, Queen's Medical Centre, Nottingham University Hospitals NHS Trust, Nottingham, GBR; 2 Respiratory Medicine, Queen's Medical Centre, Nottingham University Hospitals NHS Trust, Nottingham, GBR

**Keywords:** airway obstruction, biphasic, expiratory, inspiratory, paradoxical breathing, adducted, respiratory failure, stridor, vocal cord palsy, rheumatoid arthritis

## Abstract

Rheumatoid arthritis (RA) can cause a number of laryngeal manifestations; however, most of these do not cause an airway emergency. Airway obstruction due to vocal cord fixation of one or both vocal cords occurs late in the disease process of RA and can present as an inspiratory stridor. We report the case of an elderly lady who presented with acute stridor secondary to RA-induced bilateral vocal cord palsy and describe the various management options that were considered.

An 85-year-old woman presented to A&E Resus with tachypnoea, stridor, and drowsiness. An arterial blood gas (ABG) was performed which showed hypercapnic respiratory failure on 60% oxygen with blood tests revealing moderately raised infective markers and a chest X-ray displaying right lower zone consolidation. A flexible nasendoscopy was performed which demonstrated bilaterally fixed and adducted vocal cords due to bilateral cricoarytenoid joint fixation, with a rima glottidis measurement of approximately 3 mm and evidence of paradoxical breathing. The patient had been admitted with a similar presentation 18 months before, however not as severe, and once again, the bilateral vocal cord palsy had been attributed to her longstanding RA. She was stabilised with non-invasive ventilation and transferred to the acute respiratory care unit. Long-term surgical options were thoroughly discussed including tracheostomy, vocal cord lateralisation, cordotomy, and arytenoidectomy, but ultimately, these options were all deemed unsuitable for the patient and so a palliative care approach was adopted following the withdrawal of bilevel positive airway pressure.

Stridor is a late but life-threatening complication of RA that has viable surgical options of tracheostomy and static glottis enlarging procedures; however, the appropriateness of such procedures should always be correlated with the patient's current clinical status and the extent to which they may impact on the patient's quality of life.

## Introduction

Rheumatoid arthritis (RA) is an autoimmune condition that can affect the larynx in 13-75% of cases, and its presentation is usually subclinical [1]. Laryngeal manifestations of RA include oedema, myositis, and nodules; however, more significantly, airway obstruction can occur due to vocal cord fixation [2]. This is a life-threatening complication of RA that usually occurs late in the disease process and can present as an inspiratory stridor. Stridor occurs due to chronic inflammatory changes affecting the synovial membrane overlying the cricoarytenoid joints which can consequently cause ankylosis leading to the fixation and overall reduced mobility of the vocal cords [2,3]. We report the case of an elderly lady who presented with acute stridor secondary to RA-induced bilateral vocal cord palsy and describe the various surgical management options that were considered.

This article was previously presented as a poster at the 2023 British Academic Conference of Otolaryngology (BACO) International Conference on Thursday, February 16, 2023.

## Case presentation

An 85-year-old woman presented to A&E Resus with tachypnoea, stridor, and drowsiness. She had a medical background of coronary artery bypass graft, tissue aortic valve replacement, heart failure, chronic left pleural effusion secondary to previous left empyema, RA being managed with methotrexate, total hip replacement, and thoracic compression fractures. She had also contracted coronavirus disease 2019 (COVID-19) infection six weeks before which was initially thought to be the diagnosis on this admission. Baseline observations were conducted which revealed oxygen saturations of 99% on 15 litres of oxygen via a non-rebreather mask, a respiratory rate of 45 breaths per minute, a heart rate of 80 beats per minute, and a Glasgow Coma Scale (GCS) of 7/15. An arterial blood gas (ABG) was performed which showed hypercapnic respiratory failure on 60% oxygen: pH 7.19, pCO2 10.4, pO2 17.1, and HCO3 30.4. Her COVID-19 swab came back negative, and a recent echocardiogram revealed moderate-severe tricuspid regurgitation with non-dilated left ventricle, grade 1 diastolic dysfunction, and preserved systolic function. Blood tests showed moderately raised infective markers, and a chest X-ray demonstrated evidence of right lower zone consolidation.

A flexible nasendoscopy was performed which demonstrated bilaterally fixed and adducted vocal cords due to bilateral cricoarytenoid joint fixation, with a rima glottidis measurement of approximately 3 mm and evidence of paradoxical breathing (see Video 1). Therefore, the overall clinical impression was that of respiratory dysfunction secondary to bilateral vocal cord palsy and lower respiratory tract infection. The patient had been admitted with a similar presentation 18 months before, however not as severe, and once again, the bilateral vocal cord palsy had been attributed to her longstanding RA. She was initially stabilised with the use of bilevel positive airway pressure which helped to maintain oxygen saturations of 99% on 15 litres of oxygen; this form of non-invasive ventilation (NIV) helped to correct her hypercapnic respiratory failure, resulting in an improvement in her GCS and overall clinical state. Once stabilised, she was transferred to the acute respiratory care unit for ongoing care. 

**Video 1:** Bilateral vocal cord palsy secondary to rheumatoid arthritis

Long-term management plans were discussed at length with both the patient and her family, specifically the recommendation of a tracheostomy. However, the patient was adamant that she did not want this potentially life-saving procedure as she felt that caring for the tracheostomy long-term would be difficult and place a significant burden on her remaining quality of life, particularly since she already had extremely poor dexterity as a result of the RA. Therefore, other surgical options for this patient were discussed which included vocal cord lateralisation and vocal cordotomy/arytenoidectomy. There had already been an unsuccessful attempt at vocal cord lateralisation during a previous admission, and a consultant laryngologist opinion did not recommend any further stitch lateralisation or anatomical removal as this risked aspiration which could potentially cause more chest infections. Therefore, domiciliary NIV and palliative support as two final options were discussed with the patient and her family; the patient's wishes were for the latter, and she subsequently died a week later following the withdrawal of NIV and in receipt of best supportive care.

## Discussion

Stridor is caused by partial obstruction of the respiratory tract at the level of the larynx or trachea. Stridor can be inspiratory (supraglottic obstruction), expiratory (tracheal obstruction), or biphasic (glottic or subglottic obstruction). Stridor can be secondary to infection (supraglottitis), malignancy, trauma, anaphylaxis, neurological conditions (multiple system atrophy and myasthenia gravis), and inducible laryngeal obstruction [1]. Laryngeal involvement in RA ranges from 13% to 75%, and its presentation is usually subclinical [1,3]. Symptoms can range from degrees of dysphonia up to acute airway obstruction [4]. Specific laryngeal manifestations of RA include myositis, oedema, and rheumatoid nodules; however, these do not usually present as an airway emergency [2,5]. Fixation and immobility of the vocal cords cause airway obstruction due to chronic inflammatory changes affecting one or both of the cricoarytenoid joints [6,7]. Symptoms are found to be more severe if the cricoarytenoid joints are affected bilaterally and become permanently fixed in an adducted position [3], thereby reducing the distance of the rima glottidis through which airflow passes.

There are several surgical treatment options available for bilateral vocal cord palsy, of which the most common are tracheostomy, arytenoidectomy, and cordotomy [8]. There are other management strategies used to varying degrees of success such as botulinum toxin injections, laryngeal reinnervation techniques, and gene/stem cell therapy, though these therapies are still within an experimental phase. Tracheostomy is the primary effective treatment for a patient with bilateral vocal palsy as it provides immediate relief of airway obstruction and restores a patient's ventilation; however, it is often only a temporary measure, is less cost-effective when compared to endoscopic techniques, and is becoming disfavoured by patients due to the long-term care required and reduced quality of life associated with them [9,10]. Endoscopic techniques in arytenoidectomy and cordotomy provide a more cost-effective and permanent improvement in ventilation without unsightly external cosmesis; however, they are associated with common side effects such as dysphonia, dysphagia, granuloma formation, and aspiration [11,12]. Ultimately, it is of vital importance that the treatment option selected is suitable for the patient without affecting their quality of their life and future health.

## Conclusions

Stridor is a late but life-threatening complication of RA that has viable surgical options of tracheostomy and static glottis enlarging procedures; however, the appropriateness of such procedures should always be correlated with the patient's current clinical status and the extent to which these interventions may impact on the patient's quality of life.
